# Impact of Brown Rice as Adjunct on Beer Brewing

**DOI:** 10.3390/foods14122019

**Published:** 2025-06-07

**Authors:** Yufeng Wang, Xinyi Zhao, Suya Liu, Jiangyu Zhu, Yongqi Yin, Zhengfei Yang

**Affiliations:** School of Food Science and Engineering, Yangzhou University, Yangzhou 225000, China

**Keywords:** beverages, beer quality, wort, fermentation

## Abstract

The utilization of alternative cereals for brewing beer has garnered significant interest in contemporary times. The utilization of alternative cereals as adjuncts has great potential for creating novel beer flavour profiles and cost savings. Brown rice (BR) is the unpolished rice grain that retains its outer layer post-hulling and is nutritionally superior to polished rice (PR). The utilization of BR in beer production remains unexplored, with its brewing attributes in comparison to PR yet to be elucidated, probably due to the potential adverse impact on beer flavour. This study involves incorporating PR and BR as adjuncts in a 40% ratio, alongside 100% Pilsen malt (PM) beer as the control, to contrast the brewing attributes (physicochemical indicators, antioxidant attributes, volatiles, and sensory analysis). Raw material analysis results showed that BR contains starch (72.97%), protein (6.85%), fat (3.38%), and ash (1.04%). The protein content of PR (4.12%) was lower than that of BR (6.85%), attributed to the absence of bran in PR, resulting in a reduced free amino nitrogen (FAN) content in its wort. Furthermore, it was observed that 40% BR beer showed enhanced antioxidant properties (0.55 mmol TE/L for DPPH and 0.75 mmol TE/L for ABTS) in comparison to 40% PR beer (0.12 mmol TE/L for DPPH and 0.4 mmol TE/L for ABTS). The changes that occurred in volatile and sensory analysis indicated discernible modifications in beer flavour consequent to the partial substitution of barley malt with BR. These findings show BR is an appropriate brewing adjunct.

## 1. Introduction

Beer is a fermented beverage created from malt, water, and hops through yeast fermentation. Renowned for its dense foam and rich taste, beer is often referred to as “liquid bread” due to its composition of carbohydrates, proteins, vitamins, and minerals essential for human consumption. In 2024, the global beer market was assessed at USD 851.15 billion. Furthermore, the worldwide beer production for 2022 reached 1.89 billion hectoliters [1]. China’s beer market is the world’s largest, twice the size of the American market and more than five times that of Germany [2]. Scientific investigations have demonstrated that beer consumption may offer prophylactic benefits against oncological conditions, vascular ailments, and digestive system dysfunctions, while also exerting a salutary influence on renal function [3]. Studies suggest that it is healthier to be a moderate consumer of beer than to be a teetotaler or alcoholic [4]. In accordance with the German Purity Law, traditional brewing practices in Germany mandate the use of only four ingredients: water, barley malt, hops, and yeast [5]. In Japan, the taxation of beer products is determined by malt content, with lower malt concentrations resulting in reduced tax liabilities [6]. The prohibition of barley and barley malt imports into Nigeria from 1985 to 1999 precipitated the adoption and proliferation of malt substitutes, a trend that continues despite the repeal of the import ban [7]. Conversely, beer production regulations in other nations exhibit less rigorous standards. Barley malt serves as the primary ingredient in beer production, but in light of rising costs associated with transportation and energy, other grains such as rice, wheat, barley, corn, corn starch, and syrup are incorporated as adjuncts to lower expenses. The selection of adjuncts varies across regions based on locally grown grains [8]. Corn and corn syrup are commonly used in the United States due to their abundant production, while barley is a prevalent adjunct in European beer brewing. In Africa, sorghum is extensively cultivated and utilized in beer production, whereas rice, a globally common grain, is notably employed in Asian countries like China for brewing purposes [9]. Furthermore, alterations in climate and the escalation of extreme weather phenomena may exacerbate the cultivation challenges of barley in specific global locales, precipitating a contraction in the worldwide barley supply [10]. Consequently, the exploration of alternative cereal grains for beer production has experienced a notable surge in scholarly and industrial interest.

Rice is a vital staple crop globally, thriving in wet conditions where other crops struggle. Rich in carbohydrates (70–90%) and essential nutrients, rice grains are a key dietary source for billions, particularly in developing nations [11]. Brown rice (BR) refers to the rice grain that retains its outer layer after the hulling process, whereas polished rice (PR) is obtained by polishing BR to remove the outer layer. The exclusion of polishing processes results in reduced labour and production expenses, as it obviates the necessity of acquiring polishing machinery for the preparation of PR [12]. In addition, BR surpasses PR in terms of its nutritional value, serving as a rich source of vitamins, minerals, fibre, γ-oryzanol, and phytochemical compounds that have the potential to promote human health [13]. Considering its increased health benefits, various attempts have been undertaken to utilize BR as a key ingredient in the creation of a diverse array of food items, including but not limited to cakes, breads, and noodles [14]. Extensive research has been conducted on the utilization of PR as an adjunct for brewing [15]. The impact of incorporating BR into beer production remains to be explored. The limited research in this area may be attributed to the higher lipid content in BR compared to PR, which can lead to off-flavours in beer. Additionally, the elevated protein levels in BR, relative to PR, may contribute to haze formation.

The current study incorporated BR as a substitute for a portion (40%) of barley malt in the process of brewing beer. A 40% proportion was chosen as it is generally advised that the rice content in the grain matrix should not surpass this percentage unless supplemented with external enzymes [9]. Alongside this, beer variants using PR adjunct and barley malt were also produced for comparison. The distinctions among the samples concerning basic physicochemical characteristics, antioxidant attributes, volatile compounds, and sensory assessment were investigated, aiming to reveal the impact of incorporating BR on beer quality.

## 2. Materials and Methods

### 2.1. Materials

Pilsen malt (PM) (*Hordeum vulgare* L.) was obtained from Malteurop (Beijing, China). BR (*Oryza sativa* L.) was obtained from Dashilao (Liaoning, China). Saaz hops and Magnum hops were purchased from Yakima Chief Hops Inc. (Yakima, WA, USA). *Saccharomyces cerevisiae* SafAle US-05 was obtained from Fermentis (Lesaffre, France). The BC0700 starch content kit was purchased from Solarbio^®^ (Beijing, China). The other chemicals and reagents were analytical-grade.

### 2.2. Beer Brewing Process

#### 2.2.1. Grain Preparation

PR was obtained through processing BR by a polished rice machine (JM2010, Shenzhen, China). PM, BR, and PR were individually taken and then ground using a malt grinder (Model 500 high bucket grinder, Linyi, China). The BR and PR that passed through a 2 mm standard sieve were selected. The grains were combined into mixtures consisting of 0% BR (2.5 kg PM), 40% PR (1.5 kg PM and 1.0 kg PR), and 40% BR (1.5 kg PM and 1.0 kg BR). Each of the ground grain mixtures was then stored in a plastic bag at room temperature.

#### 2.2.2. Wort Preparation

Water (10 L) was incorporated into the mixed grain (2.5 kg) to initiate mashing. The mashing procedure adhered to Mayer’s method [16], involving a temperature of 45 °C for 30 min, followed by 65 °C for 45 min, and then reaching 74 °C. Subsequently, the iodine test was conducted every 15 min. Once the iodine solution retained its original colour, the time was recorded as the mashing time, which did not exceed 1 h to optimize time efficiency. Following the successful iodine test, the temperature was raised to 78 °C for 10 min. Then, the heating was halted and the dregs were washed with water at 76 °C and filtered. After filtration, the wort was boiled for 1 h, with Saaz hops (0.3 g/L) added at the outset and Magnum hops (0.3 g/L) added 40 min into the boiling process. Following boiling, the wort was adjusted to 12 °P and was cooled to 25 °C.

#### 2.2.3. Fermentation

The wort (10 L) was transferred to a 27 L FermZilla Conical Pressure Brewing Kit from Kegland, Melbourne, Australia, along with SafAle US-05 (0.6 g/L). The fermentation process occurred at 19 °C for 7 days, following which the temperature was reduced to 9 °C for 2 days to aid yeast settling and clarification. The G20-Glycol Chiller from Kegland, Australia, was utilized to control the temperature. The final beer samples were bottled and stored at 4 °C in a refrigerator.

### 2.3. Determination of Indicators

#### 2.3.1. Basic Physicochemical Characteristics

The analyses were performed mainly according to the Analytica-European Brewery Convention (EBC) [17].

Grain indicators: Protein (EBC 3.3.1), water content (EBC 4.2), extract (EBC 6.3), fat (EBC 6.10), and starch were determined by the BC0700 kit. Ash was obtained by the calcination method, in which crushed grains (3 g) were combusted at 550 °C until a greyish white colour was achieved, and then the ash was weighed after reaching constant weight.

Wort indicators: Colour (EBC 8.5), pH (EBC 8.17), viscosity (EBC 8.4), extract (EBC 8.3), FAN (EBC 8.10.1), and reducing sugar (DNS method).

Beer indicators: Alcohol content (EBC 9.2.1), pH (EBC 9.35), viscosity (EBC 9.38), original extract (EBC 9.4), real extract (EBC 9.4), fermentation degree (EBC 9.5), colour (EBC 9.6), and bitterness (EBC 9.8).

#### 2.3.2. Antioxidant Properties

The free radical scavenging activity (DPPH, ABTS radical scavenging activity) was determined according to a previous study [18]. DPPH assay: The test sample underwent dilution and was then combined with 2 mL of a 0.1 mM DPPH solution. The mixture was maintained at room temperature for 60 min. Absorbance was assessed at 517 nm, with anhydrous ethanol serving as the blank. ABTS assay: The ABTS stock solution was formulated by admixing equal volumes of 14 mM ABTS solution and 4.9 mM potassium persulphate solution, followed by a 12 h incubation period at ambient temperature. The stock solution was then diluted to achieve an absorbance of 0.7 at 734 nm. Then, 2.9 mL of the diluted stock solution was combined with 0.1 mL of the diluted beer sample and kept at 30 °C for 20 min. Absorbance was assessed at 734 nm.

#### 2.3.3. Volatile Compounds

The volatile components of the beers were identified using headspace solid-phase microextraction (HS-SPME) combined with gas chromatography-mass spectrometry (GC-MS) following a previous procedure with slight adjustments [19].

1. HS-SPME pre-treatment

The SPME sampler was introduced into the GC sampler port and incubated at 250 °C for 30 min. A 5 mL beer sample was transferred into a 20 mL headspace vial using a pipette. Subsequently, the surface of the sample liquid was submerged in a constant-temperature water bath at 50 ± 0.1 °C for 10 min. The SPME sampler with a 75 µm CAR/PDMS fibre was manually inserted into the headspace vial for 40 min of extraction, ensuring that the extraction needle was positioned as close to the liquid surface as possible without making contact. Following adsorption, the sampler was placed into the gas chromatograph sampler, desorbed for 5 min, and then removed.

2. GC-MS analysis conditions

Column: DB-WAX (30 m × 0.25 mm × 0.25 μm). Chromatographic conditions: The initial temperature was 50 °C; then, it was ramped to 90 °C at a rate of 2 °C/min, further ramped to 180 °C at 10 °C/min, and then to 220 °C at 5 °C/min, lasting for 2 min. The column flow rate was 1 mL/min. Helium served as the carrier gas, employed at a flow rate of 1 mL/min. For mass spectrometry analysis, a quadrupole mass analyzer was used with a temperature of 150 °C. The electron impact ion source temperature was set at 200 °C, the inlet temperature at 230 °C, the interface temperature at 220 °C, and the voltage at 70 eV, and the scan range was 29–450 amu.

#### 2.3.4. Sensory Analysis

The 20 mL of various beer samples, stored at 4 °C, was transferred into plastic cups and set aside. Twelve trained individuals were chosen from the school of food science and engineering of Yangzhou University for the evaluation. The sensory evaluation process was conducted with minor modifications based on previous research [20]. Aroma was scored on 6 dimensions including rice aroma, malty, estery, alcoholic, dimethyl sulphide (DMS), and aged flavour. Taste was scored on 6 dimensions including rice, malty, estery, alcoholic, bitter, and sour. Each dimension was scored on a scale of 1 to 10 based on sensory intensity, where 1 indicated the attribute was not prominent, and 10 indicated the attribute was very strong. The criteria of sensory evaluation are shown in Table S1. Each set of samples was blindly evaluated 3 times. The average score calculated represented the quality result of each item, and the sensory radar map was generated based on the experimental results.

#### 2.3.5. Statistical Analysis

All the experiments were repeated thrice in parallel. Single factor analysis of variance (ANOVA) was performed with IBM SPSS Statistics 25 software, and Tukey’s test was used for statistical testing. The difference was statistically significant (*p* < 0.05). The results were collected and plotted by Microsoft Excel and Origin 2021. The results of GC-MS analysis were analyzed by Turbo Mass version 6.1.2 processing software.

## 3. Results and Discussion

### 3.1. Basic Physicochemical Indicators and Antioxidant Properties

The grain indicators of PM, PR, and BR are presented in Table 1. PM had lower moisture in contrast to both PR and BR, predominantly attributed to its kilning stage within the malting process. The starch contents of PR and BR were significantly higher than that of PM, while the protein contents of PR and BR were lower than that of PM. These were due to differences in the structural composition of rice and barley malt [20]. The starch content of BR was significantly lower than that of PR. This was owing to the starch in rice mainly being distributed in the endosperm, coupled with minimal loss occurring during the polishing process [21]. The protein content and fat content of PR were significantly lower than that of BR. Proteins and fats are primarily present in bran, and were partially removed during the polishing process [13].

The wort indicators are shown in Table 2. The enzymatic activities of non-malted PR and BR are low, thereby necessitating an extended mashing time exceeding 30 min, contrasting with the mashing time of PM, which required less than 15 min. Under conditions devoid of gelatinization or enzyme supplementation, the mashing processes for 40% PR and 40% BR were successfully accomplished in less than one hour, an acceptable outcome. As can be found in the literature, quinoa, when used in unmalted form, should be applied at ratios up to 30% [22]. Reducing sugar is a key index in wort. No variance was observed in the reducing sugar content among the three wort samples under the condition of maintaining a wort °Plato value of 12. A study mentions that the incorporation of maize (45%) as an adjunct exhibited minor impacts on the wort’s reducing sugar concentration [23]. In a previous study, rice and Qingke substituted barley malt at 30% with 100% barley malt as the control. The three worts showed similar levels of reducing sugar when °Plato was adjusted to 14 [24]. Nitrogen content plays a crucial role in yeast fermentation. Given the restricted extracellular proteolytic capabilities of yeast, only amino acids, ammonium ions, and certain di- and tripeptides are able to be assimilated. FAN is employed in the synthesis of enzymatic and structural proteins, which are essential for yeast proliferation and other metabolic processes [9]. The incorporation of cereal adjuncts could influence the release of soluble nitrogen in the mashing stage, thereby affecting the FAN content [25]. The FAN content was notably reduced (decreased by 52.6% and 19.9%) in the worts containing 40% PR and 40% BR compared to PM wort. Among these, the wort with 40% PR exhibited the lowest FAN content, aligning with the corresponding protein levels found in the grains. A parallel pattern was noted in a previous study involving the utilization of rice in beer production. Maia et al. raised the proportion of rice adjunct up to 50%, resulting in a reduction in FAN content in the worts from 306 to 179 mg/L [15]. Another study investigated the characteristics of wort produced with 20%, 30%, and 40% corn in the grist. Their findings indicated that the wort samples exhibited a reduced level of FAN [26]. There were no significant viscosity differences among the three worts. This can be attributed to the utilization of short-grain rice in the present study, as it demonstrated lower susceptibility to viscosity concerns in contrast to medium- and long-grain rice cultivars [27].

The basic physicochemical indicators of PM, 40% PR, and 40% BR beer were given in Table 3. The alcohol content of 40% BR and 40% PR beers were significantly lower than PM beer (decreased by 22.22% and 19.69%). Previous studies have shown that the addition of rice significantly reduced the ratio of glucose/maltose, and lower glucose/maltose ratio typically resulted in lower alcohol content [28]. Furthermore, the fermentation degree of PM beer was notably higher than that of 40% PR and 40% BR beer, and the trend of fermentation degree of three kinds of beer was consistent with the content of FAN in wort (Table 2). Similarly, a decrease in the FAN level was also noted in germinated brown rice beer [18]. A decrease in FAN content would lead to a change in the physiological characteristics of yeast, and finally lead to a change in fermentation efficiency [29]. Correspondingly, the real extract of 40% BR beer was lower than that 40% PR beer, while the original extract was higher. Substitution of PM with 40% PR significantly reduced the viscosity level. Reduced viscosity may negatively impact the palate fullness of beer [30]. The inclusion of PR and BR significantly decreased the beer colour. This finding is in agreement with a previous study where including rice (30% or 60%) was reported to decrease the colour [31]. The pH values of the beer samples were close to the recommended value of 4.2–4.6 [32]. Humia et al. prepared beers with Beauregard sweet potato as an adjunct, which had a pH range from 4.0 to 4.3 [33]. Given the implementation of a consistent hopping regime, the beers exhibited comparable bitterness.

The antioxidant attributes of PM, 40% PR, and 40% BR beer are presented in Table 3. Generally, the removal of the outer bran layer could also lead to lower antioxidant activity of PR compared to BR [12]. The bran layer has a high concentration of phenolic substances, which gives it a high antioxidant capacity. The DPPH radical scavenging potency and ABTS radical scavenging activity of 40% BR beer were higher than those of 40% PR beer. A recent study demonstrated that beers produced with red rice as an adjunct had significantly better antioxidant activities [34].

### 3.2. Volatile Compounds in Beer Samples

The volatile components of the three beer samples are shown in Table S2. In total, 28, 27, and 30 compounds were identified in PM beer, 40% PR beer, and 40% BR beer, respectively. The majority of the volatiles came from the chemical family of alcohols (12 substances), with the second largest category being esters (12 substances).

Higher alcohols play an important role as organoleptic components present within beer. The content of higher alcohol compounds in PM beer was significantly lower than that in 40% PR and 40% BR beer. A lower FAN content within the wort could result in an excessive production of higher alcohols [35]. 2-Undecanol and decanol were not detected in 40% PR and 40% BR beers but were found in PM beer. These compounds could contribute to PM beer with waxy, floral, and citrus aromas. 2-ethyl-1-hexanol was detected in 40% BR beer, while it was not detected in PM beer and did not reach the threshold in 40% PR beer. It is characterized by a sweetness and lighter floral aroma. It was previously found in Chinese rice wine [36].

Ester compounds are the main source of fruity and floral aromas in beer [37]. The content of esters in PM beer was higher than in 40% PR and 40% BR beer. Ethyl laurate provided a mild fruity and floral aroma to beer, which was only detected in PM beer. Heptyl acetate and decyl acetate were detected in 40% BR beer at levels above the threshold, while they were not detected in 40% PR beer. These two compounds mainly show a pineapple-like fruity aroma.

Aldehyde and acid compounds were by-products of yeast fermentation and play a certain role in balancing the aroma of beer [38]. Nonanal was detected in both 40% PR and 40% BR beer and exceeded the threshold range, which is related to citrus and vinegar aromas. It has been previously identified in the Chinese-style traditional craft beer “Li” made with millet [39]. Styrene, which is described as having a slight spiciness, was identified in 40% BR beer but was not found in 40% PR beer. Other volatile compounds include olefins and phenols. α-Ionone was detected in PM beer, which imparts a floral aroma. 4-vinylguaiacol, which imparts a clove aroma to beer, was present in 40% BR beer. It was not identified in PM beer and did not surpass the threshold in 40% PR beer.

**Figure 1:**
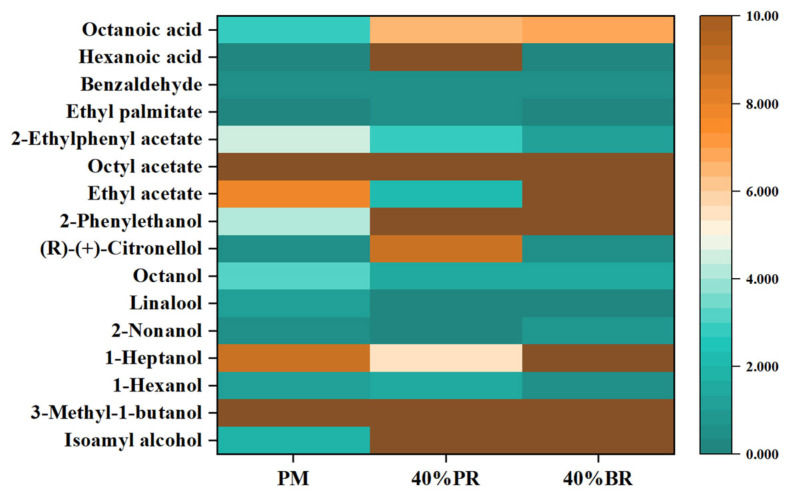
Heat map of volatile compounds in three prepared beers.

A heat map (Figure 1) was constructed for the 16 compounds present in three beers. The brown colour represents the highest standard concentration of each substance, while the blue colour denotes the lowest standard concentration. Compared with PM beer (1.828 mg/L), 40% PR and 40% BR beers had higher levels of isoamyl alcohol (19.52 and 21.311 mg/L). Isoamyl alcohol can bring a strong alcohol aroma to beer, which is the main cause of human drunkenness [40]. In another study on rice beer, isoamyl alcohol also showed a high content [41]. The contents of 2-phenylethanol in 40% PR and 40% BR beers (22.426 and 22.976 mg/L) were higher than in PM beer (17.226 mg/L), which can impart strong rose, phenol, and smoky aromas to beer. In another study, 2-phenylethanol was also considered the major compound in the volatile substances of rice beer [42]. Ethyl acetate showed higher content in 40% BR beer (11.892 mg/L), contributing to a fruity aroma and enriching the beer body, which has also been identified in other beers related to rice [43]. Octanoic acid contents in 40% PR and 40% BR beer (6.512 and 6.801 mg/L) were significantly higher than in PM beer (0.0051 mg/L). It is a common fatty acid, and the existing literature has reported that an excessive amount of fatty acids is detrimental to beer aroma [15].

### 3.3. Sensory Analysis

**Figure 2:**
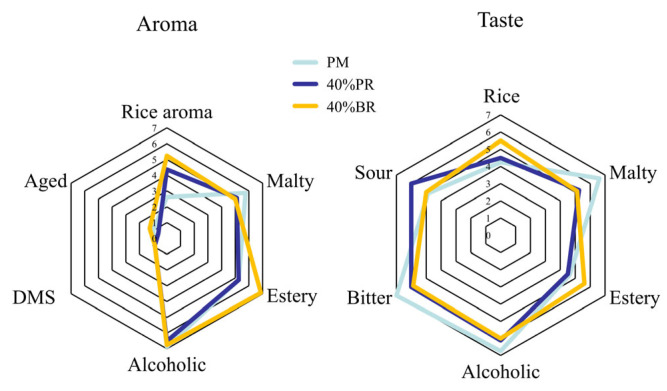
Sensory score radar map.

The sensory analysis results for the three beer samples are presented in Figure 2. The three beers received low scores for aged and DMS aroma, suggestive of their freshness and lack of off-flavours. PM beer exhibited a strong malty aroma, while both 40% BR and 40% PR beer displayed a rice aroma, with the former showing a more prominent presence. Moreover, the 40% BR beer demonstrated heightened estery and alcoholic aromas compared to the others. With regard to taste, PM beer exhibited a pronounced malty taste, whereas the 40% BR beer featured a rich rice taste and a fruity ester-like taste. This is in accordance with aroma. Mayer et al. also noted an increase in fruity flavours in rice malt beer [20]. PM resulted in beer with the highest score for bitterness. The addition of rice neutralized the bitter taste, enhancing the beer’s overall drinkability. Although there was no statistically significant difference in bitterness (Table 3), the brewing industry widely acknowledges that the distinct bitterness profiles of beers stem from various factors beyond the conventional analytical metric of bitterness units [44].

## 4. Conclusions

The present study provided information on the employment of BR as adjunct in producing beer. BR exhibited a higher concentration of nutrients, including proteins, lipids, and minerals, compared to the refined counterpart. The protein content of PR was lower than that of BR due to its lack of bran, leading to a lower FAN content in its wort. The substitution of barley malt with 40% PR or BR is feasible for mashing. The colour and alcohol levels of the beers with 40% BR were reduced compared to the 100% PM beer. Moreover, 40% BR beer exhibited superior antioxidant capabilities compared to 40% PR beer. After adding BR or PR, the content of higher alcohols increased. The main sensory benefits of BR beer over PR beer were a superior ester profile and reduced acid taste. Overall, the use of BR shows promising results for its potential use in beer brewing.

## Figures and Tables

**Table 1:** Grain indicators of PM, PR, and BR.

Indicators	PM	PR	BR
Moisture (g/100 g)	8.25 ± 0.27 ^b^	13.00 ± 0.12 ^a^	14.24 ± 0.10 ^a^
Fat (%)	1.97 ± 0.04 ^b^	2.02 ± 0.13 ^a^	3.38 ± 0.05 ^a^
Protein (%)	12.09 ± 0.11 ^a^	4.12 ± 0.63 ^c^	6.85 ± 0.11 ^b^
Starch (%)	63.65 ± 0.73 ^c^	78.99 ± 0.91 ^a^	72.92 ± 1.36 ^b^
Ash (%)	1.85 ± 0.05 ^a^	0.14 ± 0.05 ^c^	1.04 ± 0.13 ^b^
Extract (%)	75.90 ± 0.14 ^a^	68.26 ± 0.23 ^b^	72.43 ± 0.65 ^a^

Different letters in the shoulders of the same line indicate significant differences (*p* < 0.05).

**Table 2:** Wort indicators.

Indicators	PM	40% PR	40% BR
Colour (EBC)	11.82 ± 0.05 ^a^	9.35 ± 0.011 ^b^	11.22 ± 0.04 ^a^
pH	5.77 ± 0.17 ^a^	6.02 ± 0.13 ^a^	5.85 ± 0.10 ^a^
Viscosity (mm^2^/s)	1.63 ± 0.26 ^a^	1.11 ± 0.05 ^b^	1.51 ± 0.13 ^a^
Mashing time (min)	<15	30~45	30~45
Extract (g/100 g)	13.65 ± 0.14 ^a^	11.08 ± 0.10 ^b^	13.23 ± 0.15 ^a^
FAN (mg/L)	304.12 ± 0.12 ^a^	144.12 ± 0.03 ^c^	243.60 ± 0.17 ^b^
Reducing sugar (g/100 mL)	8.13 ± 0.24 ^a^	8.24 ± 0.21 ^a^	8.28 ± 0.12 ^a^

Different letters in the shoulders of the same line indicate significant differences (*p* < 0.05).

**Table 3:** Basic physicochemical and antioxidation indicators of prepared beer samples.

Indicators	PM	40% PR	40% BR
Original extract (°P)	10.80 ± 0.05 ^a^	9.77 ± 0.05 ^c^	10.18 ± 0.22 ^b^
Real extract (%)	4.65 ± 0.05 ^b^	4.83 ± 0.05 ^b^	5.38 ± 0.22 ^a^
Alcohol (%vol)	3.96 ± 0.10 ^a^	3.18 ± 0.02 ^b^	3.08 ± 0.05 ^b^
Fermentation degree (%)	58.33 ± 0.26 ^a^	51.88 ± 0.26 ^b^	48.50 ± 1.03 ^c^
Viscosity (mm^2^/s)	1.53 ± 0.11 ^a^	1.30 ± 0.09 ^b^	1.45 ± 0.11 ^a^
Colour (EBC)	11.25 ± 0.03 ^a^	7.01 ± 0.10 ^b^	7.40 ± 0.09 ^b^
pH	4.02 ± 0.01 ^b^	4.66 ± 0.04 ^a^	3.98 ± 0.18 ^b^
Bitterness (IBU)	17.75 ± 0.04 ^a^	17.35 ± 0.24 ^a^	17.15 ± 0.11 ^a^
DPPH (mmol TE/L)	0.50 ± 0.15 ^a^	0.12 ± 0.05 ^b^	0.55 ± 0.11 ^a^
ABTS (mmol TE/L)	0.78 ± 0.28 ^a^	0.40 ± 0.05 ^b^	0.75 ± 0.30 ^a^

Different letters in the shoulders of the same line indicate significant differences (*p* < 0.05).

## Data Availability

Data is contained within the article or Supplementary Material.

## Supplementary Materials

The following supporting information can be downloaded at https://www.mdpi.com/article/10.3390/foods14122019/s1: Table S1: Sensory scoring criteria; Table S2: Volatiles of three prepared beers.

## References

[1] Lee B.L., Shahin F., Selim A., Berjanskii M., Torres-Calzada C., Kovur P., Mandal R., Wishart D.S. (2024). Automated beer analysis by NMR spectroscopy. ACS Food Sci. Technol..

[2] Muhammad A., Delmond A.R., Nti F.K. (2021). Dynamic analysis of source-based preferences: The case of imported beer in China. Bri. Food J..

[3] Kaczyński P., Iwaniuk P., Hrynko I., Łuniewski S., Łozowicka B. (2024). The effect of the multi-stage process of wheat beer brewing on the behavior of pesticides according to their physicochemical properties. Food Control.

[4] Tirado-Kulieva V.A., Hernandez-Martinez E., Minchan-Velayarce H.H., Pasapera-Campos S.E., Luque-Vilca O.M. (2023). A comprehensive review of the benefits of drinking craft beer: Role of phenolic content in health and possible potential of the alcoholic fraction. Curr. Res. Food Sci..

[5] Salanță L.C., Coldea T.E., Ignat M.V., Pop C.R., Tofană M., Mudura E., Borșa A., Pasqualone A., Zhao H. (2020). Non-alcoholic and craft beer production and challenges. Processes.

[6] Brožová M., Matoulková D., Mikyška A., Kyselová L. (2022). Barley malt substitutes—Their role today and in near future. Part 1—Sugar adjuncts and barley, corn and rice as cereal adjuncts. Kvasny Prumysl.

[7] Kok Y.J., Ye L., Muller J., Ow D.S., Bi X. (2019). Brewing with malted barley or raw barley: What makes the difference in the processes?. Appl. Microbiol. Biotechnol..

[8] Donadini G., Porretta S. (2017). Uncovering patterns of consumers’ interest for beer: A case study with craft beers. Food Res. Int..

[9] Bogdan P., Kordialik-Bogacka E. (2017). Alternatives to malt in brewing. Trends Food Sci. Technol..

[10] Dabija A., Ciocan M.E., Chetrariu A., Codină G.G. (2021). Maize and sorghum as raw materials for brewing, A review. Appl. Sci..

[11] Kim S., Cho J.H., Kim H.B., Song M. (2021). Rice as an alternative feed ingredient in swine diets. J. Anim. Sci. Technol..

[12] Saleh A.S., Wang P., Wang N., Yang L., Xiao Z. (2019). Brown rice versus white rice: Nutritional quality, potential health benefits, development of food products, and preservation technologies. Compr. Rev. Food Sci. Food Saf..

[13] Mir S.A., Shah M.A., Bosco S.J.D., Sunooj K.V., Farooq S. (2020). A review on nutritional properties, shelf life, health aspects, and consumption of brown rice in comparison with white rice. Cereal Chem..

[14] Lang G.H., Kringel D.H., Acunha T.d.S., Ferreira C.D., Dias Á.R.G., Zavareze E.d.R., de Oliveira M. (2020). Cake of brown, black and red rice: Influence of transglutaminase on technological properties, in vitro starch digestibility and phenolic compounds. Food Chem..

[15] Maia C., Cunha S., Debyser W., Cook D. (2023). Impacts of adjunct incorporation on flavor stability metrics at early stages of beer production. J. Am. Soc. Brew. Chem..

[16] Mayer H., Marconi O., Regnicoli G.F., Perretti G., Fantozzi P. (2014). Production of a saccharifying rice malt for brewing using different rice varieties and malting parameters. J. Agric. Food Chem..

[17] EBC (2007). European Brewery Convention Analytica–EBC.

[18] Zhao X., Yin Y., Fang W., Yang Z. (2023). Potential of germinated brown rice in beer brewing. J. Cereal Sci..

[19] Wang S., Zhao C., Wang Y., Li C., Sun Z., Liu X., Yin Y., Yang Z., Fang W. (2022). Effects of crystal malts as adjunct on the quality of craft beers. J. Food Process. Pres..

[20] Mayer H., Ceccaroni D., Marconi O., Sileoni V., Perretti G., Fantozzi P. (2016). Development of an all rice malt beer: A gluten free alternative. LWT.

[21] Reddy C.K., Kimi L., Haripriya S., Kang N. (2017). Effects of polishing on proximate composition, physico-chemical characteristics, mineral composition and antioxidant properties of pigmented rice. Rice Sci..

[22] Kordialik-Bogacka E., Bogdan P., Pielech-Przybylska K., Michalowska D. (2018). Suitability of unmalted quinoa for beer production. J. Sci. Food Agric..

[23] Rocha dos Santos Mathias T., Moreira Menezes L., Camporese Sérvulo E.F. (2019). Effect of maize as adjunct and the mashing proteolytic step on the brewer wort composition. Beverages.

[24] Zong X., Wu J., Chen Z., He L., Wen J., Li L. (2022). Impact of Qingke (*Hulless barley*) application on antioxidant capacity and flavor compounds of beer. J. Cereal Sci..

[25] Hill A.E., Stewart G.G. (2019). Free amino nitrogen in brewing. Fermentation.

[26] Błażewicz J., Zembold-Guła A. (2007). Milled Corn products in worts production. Pol. J. Food Nutr. Sci..

[27] Cadenas R., Caballero I., Nimubona D., Blanco C.A. (2021). Brewing with starchy adjuncts: Its influence on the sensory and nutritional properties of beer. Foods.

[28] Park J., Park H.Y., Chung H.J., Oh S.-K. (2023). Starch structure of raw materials with different amylose contents and the brewing quality characteristics of Korean rice beer. Foods.

[29] Mo F., Zhao H., Lei H., Zhao M. (2013). Effects of nitrogen composition on fermentation performance of brewer’s yeast and the absorption of peptides with different molecular weights. Appl. Biochem. Biotechnol..

[30] Michiels P., Debyser W., Courtin C., Langenaeken N. (2023). Filtration enzymes applied during mashing affect beer composition and viscosity. J. Inst. Brew..

[31] Yorke J., Cook D., Ford R. (2021). Brewing with unmalted cereal adjuncts: Sensory and analytical impacts on beer quality. Beverages.

[32] Trummer J., Watson H., De Clippeleer J., Poreda A. (2021). Brewing with 10% and 20% malted lentils—Trials on laboratory and pilot scales. Appl. Sci..

[33] Humia B.V., Santos K.S., Schneider J.K., Leal I.L., de Abreu Barreto G., Batista T., Machado B.A.S., Druzian J.I., Krause L.C., da Costa Mendonca M. (2020). Physicochemical and sensory profile of Beauregard sweet potato beer. Food Chem..

[34] Santana J.C.O., Pereira de Gusmão R., Tejo Cavalcanti M., de Luna Freire K.R., Moreira de Carvalho L., Sousa Galvão M., Madruga M.S., Abrantes da Silva Souza T., Lisboa H.M., Nascimento A.P.S. (2024). The role of red rice in craft beer: A sensory and nutritional evaluation. Cereal Chem..

[35] Ceccaroni D., Sileoni V., Marconi O., De Francesco G., Lee E.G., Perretti G. (2019). Specialty rice malt optimization and improvement of rice malt beer aspect and aroma. LWT.

[36] Mo X., Fan W., Xu Y. (2009). Changes in Volatile Compounds of Chinese rice wine wheat Qu during fermentation and storage. J. Inst. Brew..

[37] Olaniran A.O., Hiralal L., Mokoena M.P., Pillay B. (2017). Flavour-active volatile compounds in beer: Production, regulation and control. J. Inst. Brew..

[38] Anderson H.E., Santos I.C., Hildenbrand Z.L., Schug K.A. (2019). A review of the analytical methods used for beer ingredient and finished product analysis and quality control. Anal. Chim. Acta.

[39] Li S., Jia J., Meng Q., Song H., Qiu R. (2024). Characterization of key odor-active compounds in Chinese-style traditional craft beer “Li”. J. Food Compos. Anal..

[40] Poisson L., Schieberle P. (2008). Characterization of the key aroma compounds in an American Bourbon whisky by quantitative measurements, aroma recombination, and omission studies. J. Agric. Food Chem..

[41] Zhang T., Zhang H., Yang Z., Wang Y., Li H. (2019). Black rice addition prompted the beer quality by the extrusion as pretreatment. Food Sci. Nutr..

[42] Lyu J., Nam P.W., Lee S.J., Lee K.G. (2013). Volatile compounds isolated from rice beers brewed with three medicinal plants. J. Inst. Brew..

[43] Zhang D., He Y., Ma C., Li H. (2017). Improvement of beer flavour with extruded rice as adjunct. J. Inst. Brew..

[44] Oladokun O., Tarrega A., James S., Smart K., Hort J., Cook D. (2016). The impact of hop bitter acid and polyphenol profiles on the perceived bitterness of beer. Food Chem..

