# Supplementary material for: Impact of Brown Rice as Adjunct on Beer Brewing
Source: Foods. 2025 Jun 7;14(12):2019. doi: 10.3390/foods14122019 (PMC12192036; doi:10.3390/foods14122019)
Supplement: Supplementary file 1 [file foods-14-02019-s001.zip › foods-3677990-supplementary.pdf]

Supplementary Materials

Impact of brown rice as adjunct on beer brewing

Table S1. Sensory scoring criteria

	Item	Aromatic Description	Reference standard
Aroma	Rice aroma	Rice aroma	Crushed rice
	Malty	Pilsen malt aroma	Crushed Pilsen malt
	Estery	Slight fruity, solvent-like aroma	200 ppm ethyl acetate solution
	Alcoholic	Ethanol aroma	50 g/L ethanol solution
	DMS	Cardboard and dimethyl sulfide aroma	100 μg/L DMS solution
	Aged	Stale breadcrumbs and aged beer aroma	25 mg/L 5-methylfuran solution
Taste	Rice	Taste of rice	Rice wort
	Malty	Taste of Pilson malt	Pilson malt wort
	Estery	Taste of fruits	Fruit juice
	Alcoholic	Taste of ethanol	Commercial baijiu
	Bitter	Taste of caffeine	Commercial coffee
	Sour	Taste of white vinegar	Commercial white vinegar

Table S2. Volatiles of three prepared beers

Classification	RT/min[1]	Compound	Odour threshold[3] (mg/L)	Content（mg/L）			Description[2]
				PM	40% PR	40% BR	
Higher alcohols	5.98	Isoamyl alcohol	0.033	1.828	19.520	21.311	5.98
	9.51	3-Methyl-1-butanol	0.0061	64.701	78.382	79.621	9.51
	15.83	1-Hexanol	0.034	1.146	1.011	0.619	15.83
	21.01	1-Heptanol	0.023	8.990	5.500	11.793	21.01
	22.07	2-Ethyl-1-hexanol	0.8	ND[4]	0.477	3.550	22.07
	23.06	2-Nonanol	0.058~0.082	0.476	0.117	0.071	23.06
	23.66	Benzyl alcohol	0.0024	1.253	0.183	0.267	23.66
	23.92	Normal octanol	0.022	2.376	1.119	1.278	23.92
	24.44	Decanol	0.0050	0.056	ND	ND	24.44
	26.75	2-Undecanol	0.0086~0.041	0.343	ND	ND	26.75
	27.36	(R) - (+)-β-citronellol	0.04~0.05	0.343	0.166	0.525	27.36
	29.12	2-Phenylethanol	0.012~0.021	17.226	22.426	22.976	29.12
		Subtotal		98.738	128.901	142.011	

	2.61	Ethyl acetate	0.88	6.239	2.321	11.892	Floral and fruity
	6.02	Isoamyl acetate	0.067~0.918	31.082	25.954	27.214	Banana
	6.35	Amyl acetate	2.2~2.7	1.603	0.958	ND	Banana
	10.14	Hexyl ethyl ester	18.1~27.4	29.993	18.443	13.394	Fermented flavor, fruity
	14.23	Heptyl ethyl ester	0.24	1.655	ND	1.391	Pineapple
	16.36	Ethyl heptanoate	6.3	1.905	0.628	0.353	Floral and fruity
	19.66	Octyl acetate	0.040	40.450	19.585	22.458	Floral and fruity
Esters	25.49	Decyl acetate	0.53	0.776	ND	1.557	Fruity
	26.31	Trans-2-decanoate ethyl ester	-	ND	0.707	1.676	Fruity
	27.95	2- Ethyl phenyl acetate	3.8	4.367	2.966	1.238	Rose and fruity
	28.44	Ethyl laurate	0.002	0.231	ND	ND	Mild fruity and floral
	33.53	Ethyl palmitate	>14	0.100	0.499	0.296	Waxy, fruity, cheesy
		Subtotal		118.401	72.061	81.469	

Others	10.81	Styrene	0.25	9.653	ND	6.200	Slight spicy
	16.96	Nonanal	0.0031	ND	0.191	0.758	Citrus and vinegar
	22.56	Benzaldehyde	0.085	0.550	0.405	0.411	Cherry
	24.90	Isobutyric acid	-	ND	0.616	0.064	Irritant
	26.38	3-hydroxylauric acid	-	ND	0.152	0.019	Mild rose flavor
	25.88	α-Ionone	0.16	0.407	ND	ND	Floral
	28.61	Nerylacetone	-	ND	0.852	1.892	Orange flavor
	28.85	Hexanoic acid	0.0048	2.172	22.129	0.112	Fruity, cheesy
	31.42	Octanoic acid	0.0051	0.046	6.512	6.801	Sour
	32.49	4-Vinylguaiacol	0.294	ND	0.072	0.484	Clove
	34.09	2,4-di-tert-butyl phenol	-	0.132	0.088	0.425	Mild phenolic
		Subtotal		12.960	31.017	17.166	

Note: 1. Retention time was retention index on Thermo-TG-WaxMS capillary column.

2. Flavor description come from https://www.flavornet.org and reference.

3. Odour threshold was referred from the book Compilations of odour threshold values in air, water and other media.

"-" indicates no data found.

4. "ND" indicates that it is not detected, and the experimental results are the average of three parallel experiments.
